# Polymeric Implants for the Treatment of Intraocular Eye Diseases: Trends in Biodegradable and Non-Biodegradable Materials

**DOI:** 10.3390/pharmaceutics13050701

**Published:** 2021-05-12

**Authors:** Paulina García-Estrada, Miguel A. García-Bon, Edgar J. López-Naranjo, Dulce N. Basaldúa-Pérez, Arturo Santos, Jose Navarro-Partida

**Affiliations:** 1 Departamento de Ingenieria de Proyectos-CUCEI, Universidad de Guadalajara, C.P. 45157 Zapopan, Mexico; paulinagestrada@gmail.com (P.G.-E.); garciabon.miguel@gmail.com (M.A.G.-B.); edgar.lopezn@academicos.udg.mx (E.J.L.-N.); dulcebasaldua.0528@gmail.com (D.N.B.-P.); 2 Tecnologico de Monterrey, Escuela de Medicina y Ciencias de la Salud, Campus Guadalajara, C.P. 45138 Zapopan, Mexico; asantos@e-retina.com

**Keywords:** polymers, intraocular implants, implant biocompatibility, biodegradable materials

## Abstract

Intraocular/Intravitreal implants constitute a relatively new method to treat eye diseases successfully due to the possibility of releasing drugs in a controlled and prolonged way. This particularity has made this kind of method preferred over other methods such as intravitreal injections or eye drops. However, there are some risks and complications associated with the use of eye implants, the body response being the most important. Therefore, material selection is a crucial factor to be considered for patient care since implant acceptance is closely related to the physical and chemical properties of the material from which the device is made. In this regard, there are two major categories of materials used in the development of eye implants: non-biodegradables and biodegradables. Although non-biodegradable implants are able to work as drug reservoirs, their surgical requirements make them uncomfortable and invasive for the patient and may put the eyeball at risk. Therefore, it would be expected that the human body responds better when treated with biodegradable implants due to their inherent nature and fewer surgical concerns. Thus, this review provides a summary and discussion of the most common non-biodegradable and biodegradable materials employed for the development of experimental and commercially available ocular delivery implants.

## 1. Introduction

**Figure 1:**
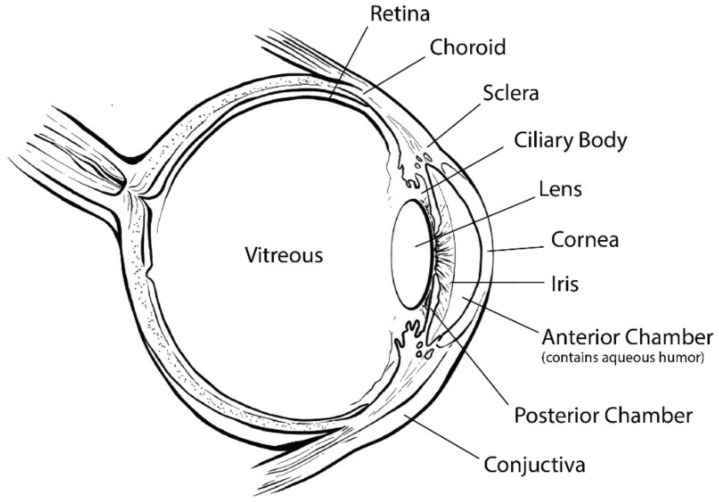
Schematic of the eyeball structure.

The eye is one of the most sophisticated and important parts of the human body, and allows us to be aware of the world around us. The eye can be divided in two segments: anterior and posterior. The anterior segment comprises tear, cornea, conjunctiva, anterior and posterior chamber, iris, ciliary body, lens, and aqueous humor, whereas the posterior segment is formed by sclera, choroid, retina, and vitreous humor [1] (Figure 1).

All the parts that constitute the entirety of the eye act as natural barriers for several drugs and proteins, making drug delivery into the eye a difficult task. For example, topically administered drugs show a bioavailability less than 5% due to the existence of different dynamic and static obstacles, being even two to three orders of magnitude lower in the posterior segment, where the retina is located. These low absorption levels are due to the existence of natural anatomical and physiological barriers. From all existing ocular barriers, the main one according to the routes of administration is tears, which are a pre-corneal film consisting of three layers with a thickness of approximately 3 μm. Tears represent the first and most prominent obstacle that drugs encounter if their administration is topical. On the other hand, the cornea is a transparent and avascular part of the eye that represents a natural barrier for both hydrophobic and hydrophilic drugs due to the composition of its constituent layers. The conjunctiva, which is a fibrous part of the eye that covers one-third of the entirety of the eyeball, is also an important obstacle that does not allow drugs between 20 and 40 kDa to pass through. Regarding the sclera, although its permeability is under debate, recent studies showed that it is not strictly a barrier, because molecules with a molar mass up to 150 kDa are able to penetrate it. Migration through the sclera is possible due to its fibrous nature that allows the existence of large pores. Finally, the vitreous chamber, which consists of a semisolid structure containing a mixture of fluids called vitreous humor, was also considered as a drug barrier because drugs can be dragged during the renovation process of the fluids. Dynamic barriers (e.g., tears, vitreous humor) produce drug drainage into the systemic circulation, while static factors (e.g., cornea, conjunctiva) retard drug absorption in the ocular tissues. Secondary factors such as blinking are also involved in the reduction of the absorption of topical drugs [1,2,3,4,5,6,7].

The intravitreal route, which is related to the use of injections and implants, is a common option to release drugs into the posterior segment of the eye. The use of injections allows a quite rapid uptake since a direct insertion of the drug into the vitreous chamber occurs. However, they are associated with adverse effects due to the penetration of a needle into the eyeball. In addition, to maintain an effective drug concentration, repeated injections are required, causing clinical complications and/or patient discomfort. The existence of natural barriers that jeopardizes drug bioavailability gave way to the design of intravitreal and periocular implants, which allow supplying drugs in a controlled and sustained way, maintaining a therapeutic concentration for a prolonged period of time. Regarding their biodegradability, implants can be classified in two categories: biodegradables and non-biodegradables. Biodegradable implants (BI) are made of polymers that show the following properties: ability to be metabolized in the body and eliminated by a physiological pathway, ease of fabrication, and ability to break down into nontoxic materials and avoid inflammation after application. BI are the most suitable option for drug delivery since only a single surgical procedure is required. Furthermore, the residues of the degradation process are expected to be carbon dioxide, water, and/or minerals. On the other hand, non-biodegradable implants (NBI) are made of materials that are not affected by the surrounding biological environment and do not experience changes in their structure throughout the treatment period. Consequently, NBI require two surgical procedures, one for implantation and a second one for removal [2,8,9,10,11,12,13]. In this regard, in the present work, a detailed summary on the polymers used in NBI and BI is provided.

## 2. Nonbiodegradable Implants

**Figure 2:**
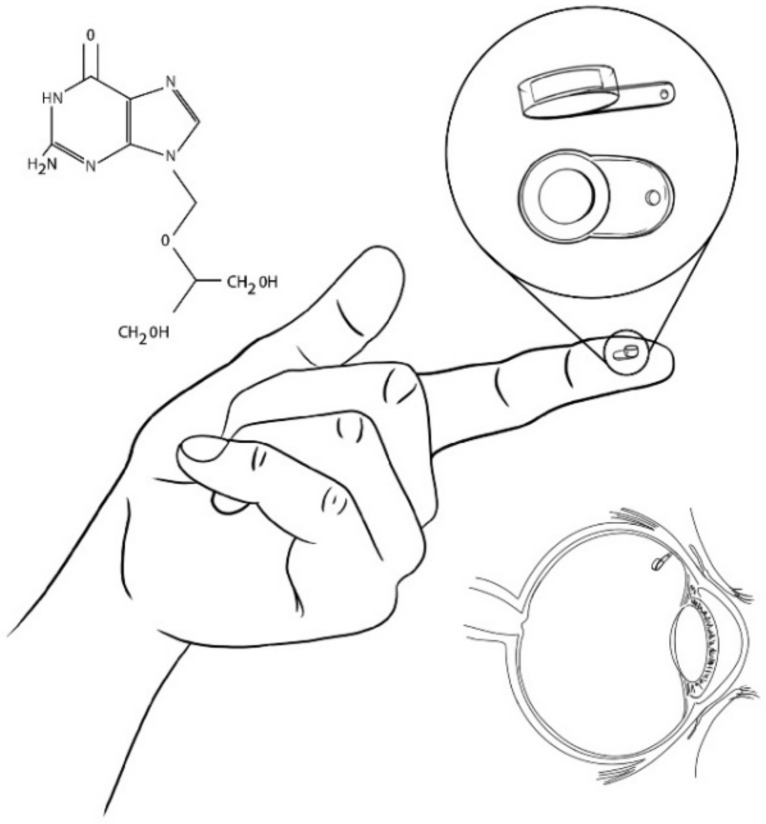
Vitrasert (Ganciclovir-loaded implant).

Owing to the disadvantages in the administration methods of ganciclovir for the treatment of cytomegalovirus retinitis, in 1992, an intravitreal device named Vitrasert made of ethylene-vinyl acetate (EVA) coated with polyvinyl alcohol (PVA) was successfully developed. Vitrasert was the first implantable intravitreal device made of a non-biodegradable polymer (Figure 2). Since its appearance, Vitrasert achieved its permanence in the market due to a superior control of retinitis over systemic ganciclovir therapy. NBI typically consist of drug release in membranes that surround a reservoir entrapping a drug. The membrane releases the drug at predetermined periods, guaranteeing a prolonged administration. Depending on the device size and the duration of the treatment, NBI could require multiple surgeries to be implanted, removed, and re-implanted as necessary. Therefore, treatments are expensive and cause patient noncompliance. Furthermore, due to the use of non-biodegradable polymers and to possible post-surgery complications, side reactions in the eye such as endophthalmitis, pseudoendophthalmitis, vitreous haze, hemorrhage, cataract development, and retinal detachment can occur [8,9,14,15,16,17,18].

EVA, PVA, polyurethanes (PU), and polysiloxanes (PS) are among the most relevant non-biodegradable materials that exist in the market due to their excellent mechanical and physical properties and their proven biocompatibility, which is understood as the ability of a material to perform with an appropriate host response in a specific application [14,19].

## 3. EVA-PVA

**Figure 3:**
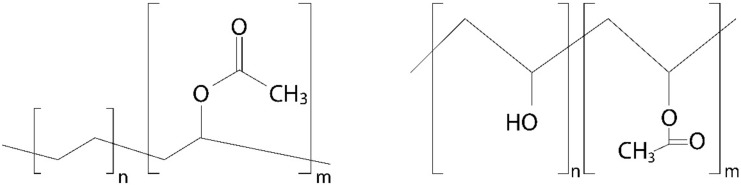
Chemical structures of EVA (left) and partially acetylated PVA (right).

EVA is a transparent thermoplastic, ethylene monomer copolymer (Figure 3) that normally contains from 1 to 40% of vinyl acetate (VA) monomer. Depending on the percent of VA, EVA may take different physical properties; the more VA, the higher polarity, adhesion, impact resistance, flexibility, and compatibility with other polymers. However, when increasing VA, a decrease in crystallinity, stiffness, softening, and melting point is also observed, as exemplified in Table 1 and Table 2 [20,21]. The probability of modifying most of the properties of EVA regulating VA content offers a broad range of possibilities in the design of new materials. EVA is a highly versatile material and one of the most used non-biodegradable polymers for the development of implantable controlled drug-release devices. Its biocompatibility is widely documented and includes information regarding cytotoxicity, irritation, and implantation into the eye and brain. EVA is a Food and Drug Administration (FDA) approved material for indirect contact and belongs to the list of inactive ingredients for approved non-drug products [22,23].

On the other hand, PVA is a permeable synthetic polymer (Figure 3) based on petroleum resources; its final properties depend on the characteristics of its parent polymer, polymerization conditions, and the degree of hydrolysis [16,24]. PVA’s applications are endless, as in textile, paper, and ceramic industries [25]. PVA is also often used in the medical field and more specifically in ophthalmology to produce contact lenses, eye drops, hydrogels, and implants; as a permeable polymer, it can act as the framework of intraocular implants regulating the rate of drug release [26,27].

As stated before, the first non-biodegradable intraocular device was made of EVA-PVA; in this device, EVA was used as a membrane to limit the surface area for the permeability of the medication, while PVA was used to build the frame of the device and to regulate the rate of drug permeability. In vivo tests revealed that the polymer-based device was well tolerated intraocularly and that it did not experience significant biodegradation. Additionally, before Vitrasert was approved by the FDA in 1996, a biocompatibility test of EVA in rabbits’ corneas was performed to measure the inflammation that could be caused in the cornea; related outcomes indicated that alcohol-washed EVA had the best results of all polymers tested, since it did not cause any observable inflammations, while unwashed EVA caused a 60% visible mild inflammation [28,29]. Although there are no recent studies of the biocompatibility of EVA in the eye, the continuous use of this copolymer in eye implants has shown that there are no clinical toxic effects [30].

PVA biocompatibility with the eye was first tested in vivo by Olson et al. [31] implanting intraocular lenses in cats; according to whole-eye histopathologic studies, no clinical signs of unusual inflammation or toxicity were observed. After being used for the first time in Vitrasert, PVA was employed as a coating material in other devices such as Retisert and Medidur [32].

**Figure 4:**
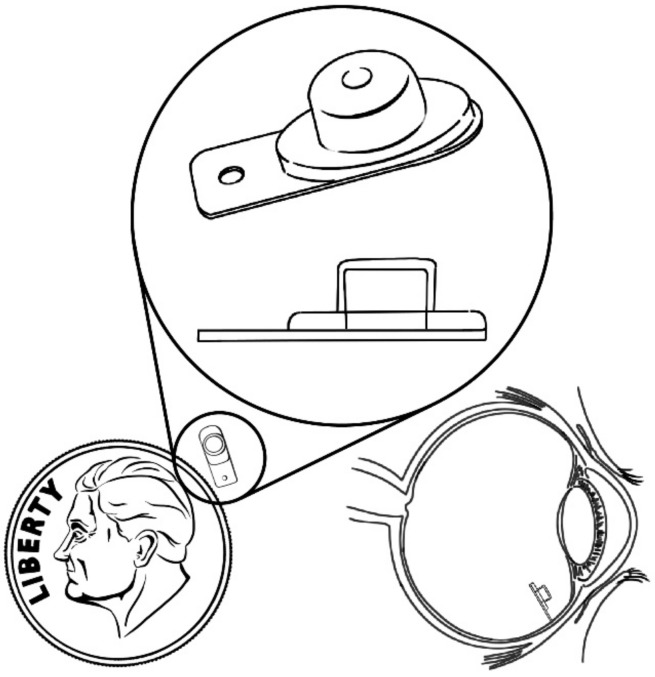
Retisert (intravitreal implant).

Retisert, a sterile intravitreal implant (Figure 4) that is similar in size to a grain of rice, was designed to release fluocinolone acetonide (FA) to the posterior segment of the eye for the treatment of chronic noninfectious uveitis, which is the fifth most common cause of vision loss and accounts for 5–20% of legal blindness. This device was approved in 2005 and was the second FDA-accepted NBI (Figure 4). Retisert shows a tablet geometry and consists of FA attached to a PVA suture tab coated with PVA and silicone layers; it is surgically implanted into the vitreous humor through a pars plans incision and anchored to the sclera by a suture. Retisert releases the drug for a period of three years while maintaining the anti-inflammatory effect after a single implantation. It was successfully used to reduce uveitis recurrence, improving visual acuity and reducing the need for adjunctive therapy, especially in patients who do not respond to or are intolerant to conventional treatments. Potential problems associated to the use of this implant include the dissociation of its main components, which could lead to vision problems, removal complications, and hemorrhage. Additionally, patients who used these devices required subsequent cataract surgery and treatment for intraocular pressure [16,17,33,34,35,36,37,38,39].

**Figure 5:**
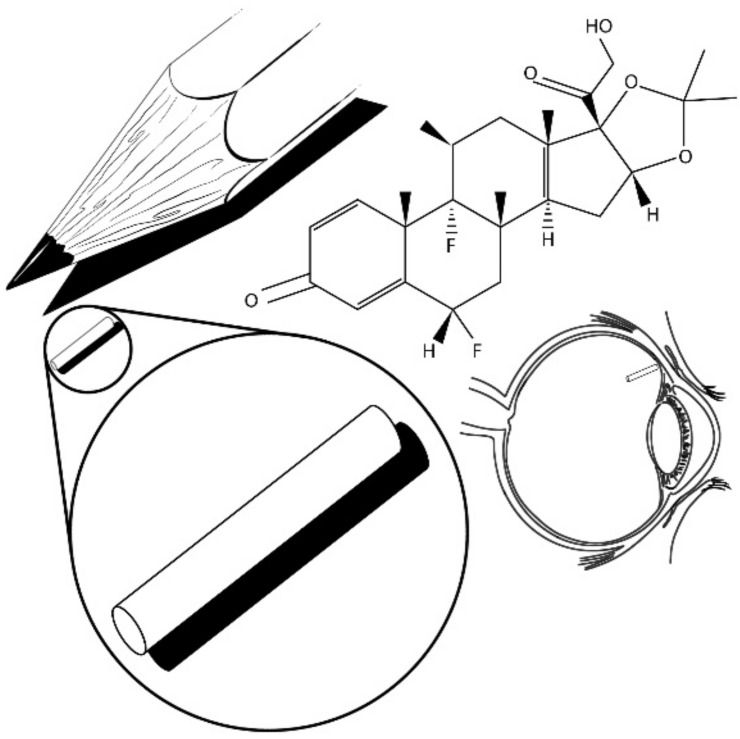
Medidur (FA-loaded device).

On the other hand, Medidur (Figure 5) consists of FA surrounded by a cylinder with PVA end caps which regulate the drug release rate. Safety studies demonstrated that these devices are well tolerated since no observable inflammation, neovascularization, fibrin, or vitreous opacity were detected [40].

Recently, Molokhia et al. [41] developed a refillable PVA matrix-based intraocular implant for drug delivery using polymethylmethacrylate (PMMA) sheets as reservoir material and silicone check valves for refilling. Unlike Vitrasert, Retisert, and Medidur, which require intravitreal procedures and suturing, and are non-refillable devices, this novel intraocular device fits in a lens capsule, and is refillable and able to deliver multiple drugs. In this implant, PVA was successfully used as a polymeric carrier to achieve a controlled release and stability of the drug.

**Figure 6:**
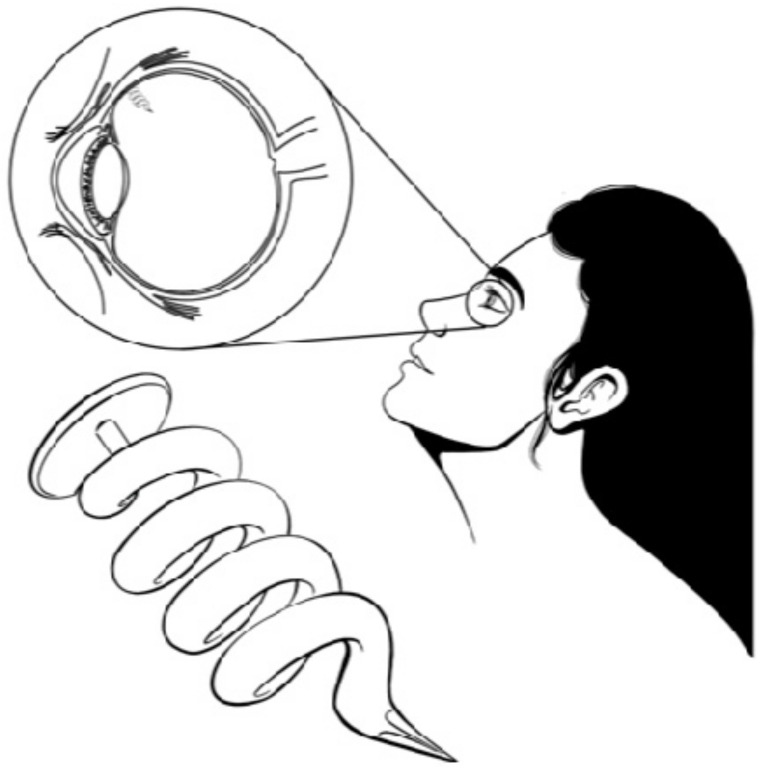
I-vation (helical-shaped implant).

Another commercially available device is I-vation (Figure 6), which is a helical-shaped implant containing triamcinolone acetonide (TA) inside a titanium-PVA-EVA reservoir. This device can release the drug for up to two years, and its design increases the surface area for drug delivery, favoring drug diffusion [14,42]. Finally, Iluvien is a cylindrical tube-shaped intravitreal device similar in composition to Retisert, with one end of the tube coated with PVA and the other end with a silicone adhesive that delivers FA to the retina up to a maximum of 36 months for the treatment of diabetic macular edema (DME). Iluvien is the smallest NBI, which allows it to be injected with a 25-gauge needle, creating a self-closing hole. This device was demonstrated to improve patient vision efficiently for up to 36 months. However, a complete treatment could require more than one application. Recent studies indicate that supplementary treatments were administered more than once after 12-month periods. Nevertheless, multiple devices can remain implanted without requiring subsequent removal due to Illuvien’s size. Compared to Retisert, Iluvien could have a more favorable side-effect profile due to the position of the implant relative to the ciliary body and/or the trabecular meshwork [14,35,38,43,44,45].

## 4. PS

**Figure 7:**
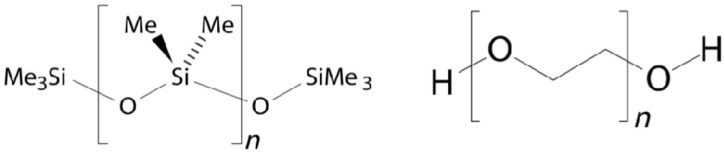
Chemical structures of PDMS (left) and PEG (right).

PS or silicones show thermal stability, biocompatibility, chemical inertness, and elastomeric properties that can be exploited to developed ocular implants. Silicones used in medicine are vulcanized using poly-dimethylsiloxanes (PDMS) (Figure 7) and a platinum compound as a catalyst [46]. PDMS is a biostable hydrophobic synthetic material with properties such as biocompatibility, nontoxicity, transparency, durability, and bioinertness that inhibits microbial growth [47,48,49]. PDMS has been used to produce membranes to control drug delivery rate and implantable devices for drug delivery [50]. For example, Lee et al. [51] developed an implantable device consisting of a PDMS-based reservoir and different configurations of micro/nanochannels embedded between two covers to simulate drug diffusion rates according to Fick’s Law, which indicates that diffusion will take place from a high concentration area to a low one.

## 5. PU

**Figure 8:**
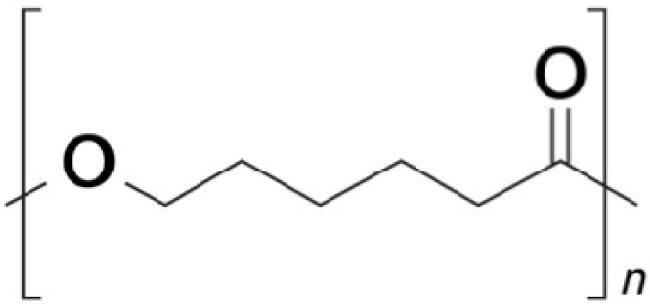
Chemical structure of PCL.

PU are rubber-like materials with a transition temperature lower than room temperature that show biocompatibility, fatigue resistance, and durability. PU generate potentially toxic by-products and are considered non-degradable materials, due to a poor degradation behavior. However, elastomeric biodegradable PU can be prepared by incorporating labile hydrolysable moieties in the PU backbone; in this case, the presence of terminal hydroxyl groups allows for alternating blocks (i.e., segments) in the PU chain. Hard segments improve hardness, tensile strength, impact resistance, stiffness, and modulus. On the other hand, soft segments increase water absorption, elongation, elasticity, softness, and degradability. Polyethylene glycol (PEG) (Figure 7) and polycaprolactone (PCL) (Figure 8) are frequently used to improve the biodegradation and polymer solubility in water and to generate non-toxic degradation by-products [52,53,54,55,56]. The effect of different segments on the properties of PU is listed in Table 3 [56]. The above-mentioned modifications to the PU main chain have allowed for the fabrication of permanent implantable devices. For example, ocular implants to treat uveitis loaded with DXM using biodegradable PU-containing clay nanoparticles (CNPs) and PCL oligomers as soft segments were produced in 2011. The use of CNPs allowed for the modification of mechanical properties in order to mimic soft ocular tissues, to keep stiffness during the biodegradation process, and to produce devices rigid enough to resist a medical procedure. These devices were tested in vitro using human retinal pigment epithelial cells demonstrating to be non-toxic or to produce toxic components [57]. TA has also been loaded in PU devices, in this case for the treatment of ocular disorders. In this work, PCL oligomers were used as soft segments [58]. In 2013, Silva Paula et al. [59] developed PU-subconjuctival implants loaded with bevacizumab, which were well tolerated in rabbit eyes and reduced the number of vascular endothelial growth factors expressing fibroblasts.

## 6. Materials Trends in NBI

**Figure 9:**
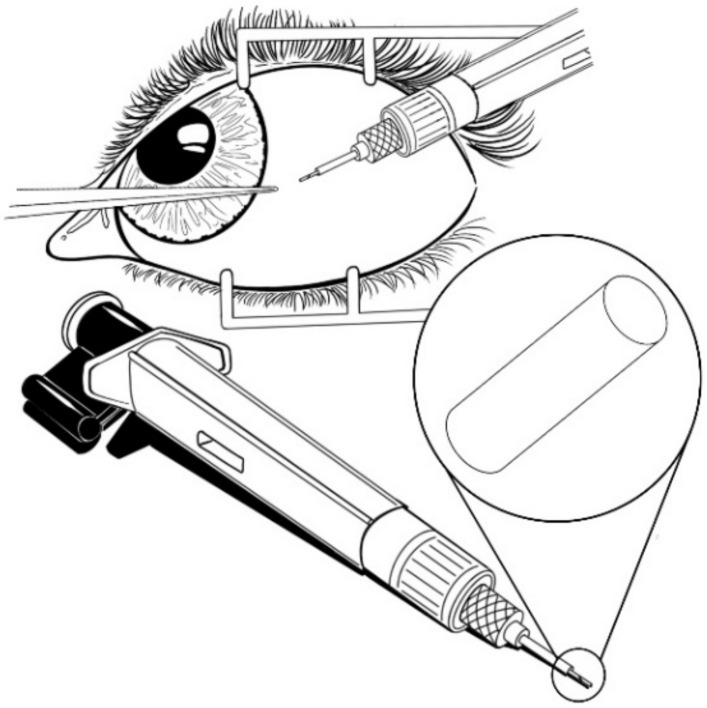
Yutiq (FA-loaded device).

At this point, it is clear that NBI basically consist of reservoir and matrix systems that control the diffusion of the drug into the body. So far, EVA, PU, and PS are the most frequently used materials for the design of ocular devices due to their favorable physical/mechanical properties and biocompatibility [60]. However, in 2018, the FDA approved a novel device containing FA called Yutiq (Figure 9), which is nearly identical to Iluvien and was designed to offer a better performance than Retisert in the treatment of uveitis [61]. In contrast to Retisert, which is implanted via surgery, Yutiq is an injectable device [62]. According to the manufacturer, the device consists of an impermeable polyimide (PI) tube with a permeable PVA membrane at one end and an impermeable silicone adhesive plug on the other end [63]. Besides commercially available devices, there are current research works that are based on the development of new implants using PI. For example, Cai et al. [64] reported on a PI tubular laboratory device loaded with FA, having one end capped with PVA and the other with either PVA or silicone. The device was shown to maintain a therapeutic effect for 36 months. Results suggested a comparable if not better inflammation control compared to widely known devices such as Retisert and Ozurdex.

PIs, particularly aromatic ones, were tested for biomedical applications; these materials are characterized by being thermally stable materials (T > 500 °C) and good light transmitters, as well as demonstrating chemical resistance (even to organic solvents), with high mechanical strength and a high modulus that can be shaped into different designs (e.g., films foams, etc.). In addition, they have proven to be biostable and functional for months in chronic in vitro and in vivo studies, as well as non-toxic by multiple in vitro, direct, or indirect cytotoxicity assays, which makes them able to be used in encapsulating implant devices [65,66]. Some relevant properties of PIs are shown in Table 4 [67].

## 7. Biodegradable Implants

Biodegradation refers to an enzymatic or non-enzymatic hydrolysis of the polymeric backbone chain, typically used in products that are water soluble or are metabolized. During biodegradation, two main processes, denominated degradation and erosion, take place. The former is related to the breakage of the main chain into low molecular polymeric fractions, while the latter refers to the process of conversion of a hydrophobic polymer into a water-soluble material. This process is closely related to the permeability of the polymer to water and the rate at which erosion of the polymer takes place. Commonly, the process is classified into two categories: surface-erosion (i.e., polymer erodes slower than water penetrates it) and bulk erosion (i.e., water penetrates into the polymer faster than polymer degradation takes place). Biodegradable polymers used to build ocular implants also offer favorable mechanical properties and the possibility of adjusting degradation rates through the manipulation of molar mass, composition, conformation, and crystallinity of the polymers [12,68].

The use of biodegradable devices fabricated using polymers, which can degrade into nontoxic byproducts or polymers that can be solubilized in vivo and eliminated safely from the human body, constitutes a way to ensure eye health. The design of biodegradable implants is a major task due to regulatory requirements, complexity, and the high cost of polymers that show suitable degradation kinetics and mechanical properties. The process to approve a biodegradable implant should take into consideration different aspects such as the age of the patient, changes in the body’s anatomy, and physiological properties that may influence the performance of the device. Biodegradable devices can be shaped into rods, plugs, pellets, discs, and sheets by means of compression molding, extrusion, or solution casting. The main advantage of biodegradable devices lies in the fact that they do not need to be removed after their useful life. On the other hand, the main disadvantages of release systems using biodegradable polymers include the so-called final burst stage of the device that occurs when hydrolysis of the polymer reaches a critical point and can lead to an uncontrolled release of the remaining drug load. Additionally, BI can cloud vision, and their movement into the anterior chamber or in front of the retina can be a complication. In order to reduce the negative impact of this final stage, polymers with different molar masses are blended to obtain a more stable release agent [10,11,13,16,68,69,70].

Biodegradable devices are typically fabricated using polycaprolactone (PCL), polylactic acid (PLA), polyglycolic acid (PGA), and poly(lactic-*co*-glycolic-acid) (PLGA) [9,10].

## 8. PCL

PCL is an aliphatic compound that is hydrophobic, semicrystalline, and highly soluble in chloroform, benzene, toluene, and other organic solvents; it is a polyester composed of hexanoate repeat units able to biodegrade due to the hydrolysis of its ester linkages under physiological conditions within several months to years depending on the molar mass, the degree of crystallinity, and the degradation conditions, which allows for well-controlled and long-term drug release periods. PCL shows a bioresorbable and biodegradable nature and due to its mechanical properties, it can be easily molded into different shapes (e.g., thin films, capsules, and others) [70,71,72,73,74,75,76]. Main properties of PCL are indicated in Table 5 [77].

So far, different experimental devices using PCL have been developed. Kim et al. [74] developed intracameral PCL implants loaded with a proprietary ocular hypotensive agent (DE-117) for the treatment of glaucoma. In vitro studies showed that the devices were well tolerated in the intracameral space with a sustained drug release rate of 0.5 g/day over six months. Analysis of the device demonstrated that DE-117 was well protected from hydrolysis, since the concentration of its hydrolyzed active form was below the limit of detection. Ocular biocompatibility of PCL films was also evaluated. For example, spin-casted nanostructured PCL films implanted using a 20-gauge needle injection were analyzed to determine their ocular tolerance by means of serial toxicity ophthalmic studies performed after 1 day, 1–4 weeks, and 1–6 months, and showed an acceptable ocular safety profile in all cases. Results also indicated that nanostructured PCL films were kept up to nine months, and no substantial cellular debris, inflammatory cells, or fibrosis was observed on the device surface [75]. Hybrid PCL thin-film devices obtained from the combination of microporous and nonporous PCL were fabricated by sealing films together with heat for long-acting protein delivery into the back of the eye. PEG was used in the device reservoir to prevent protein access to the solvent. Consequently, protein stability improves by sequestering the majority of the protein in the solid state and limiting its maximum soluble concentration within the reservoir. Devices were positioned in the anterior vitreous for a period of 84 days. Fundus photography and retinoscopy were used to evaluate signs of adverse reaction. Results showed no signs of retinal trauma, vascular congestion, or tortuosity. An in vivo study demonstrated tolerability in non-human primates for up to 12 weeks [78]. In addition, PCL spray-coated films loaded with TA were surgically implanted to evaluate their performance as an alternative to conventional subtenon TA injection for the treatment of chorio-retinal diseases. Episcleral implants did not show any adverse effect (i.e., inflammation or infiltration) during a three-month observation, which could be due to the fact that PCL does not generate an acidic environment when it degrades, unlike other polymers such as PLA [79].

Additionally, Borhani et al. [80] evaluated the efficiency of a device designed using high molar mass PCL containing 5-fluorouracil (5-FU) for the treatment of proliferative vitreoretinopathy (PVR), and obtained statistically significant results in the prevention of PVR. In 2005, a corticosteroid TA-loaded PCL subretinal implant was developed to overcome the existing limitations in the treatment of retinal diseases, and results indicated that the implant eluted steroids for a period of at least four weeks without producing an inflammatory response or complications [81]. Later, the performance of a PCL intravitreous implant loaded with dexamethasone (DXM) was studied by Silva-Cuhna et al. [82], who observed that the implant delivered the drug in a controlled and prolonged way during at least 55 weeks, after which time 79% of the drug was still present in the implant due to a slow PCL degradation process.

PCL copolymers have also been used as carriers using different drugs for the treatment of ocular diseases. For example, chronic uveitis was experimentally treated using white rabbits’ eyes and cyclosporine A contained in a glycolide-*co*-lactide-*co*-caprolactone copolymer (PGLC) device, and results indicated that intraocular inflammation was effectively reduced in rabbits without causing toxicity, providing a safe and convenient treatment [83]. PGLC was also used to release FK506 into the anterior chamber for the prolongation of corneal allograft survival in high-risk keratoplasty, and a sustained drug release was observed for at least 168 days with no adverse reactions, effectively preventing immune rejection; the mean graft survival time was also improved up to >180 days [84].

Finally, the effect of porosity was also evaluated; in 2019, intraocular implants were developed using porous poly(ε-caprolactone) in order to obtain implants that degrade faster than those produced using common PCL. Porous implants prepared via a green supercritical carbon dioxide foaming/mixing method (SFM) showed a higher surface area that allows for a faster degradation of the device under alkaline conditions and a higher release rate of the tested drug. The porosities produced by the SFM method seem to increase hydrolytic and enzymatic degradation rates of poly(α-esters) such as PCL. In addition, no cell death was observed, indicating that the devices are not toxic to retinal cells [85]. Although PCL was utilized in several experimental intravitreous, subretinal, and intracameral implants [74], no PCL-base device has been brought yet to the market.

## 9. PLA, PGA, PLGA

**Figure 10:**
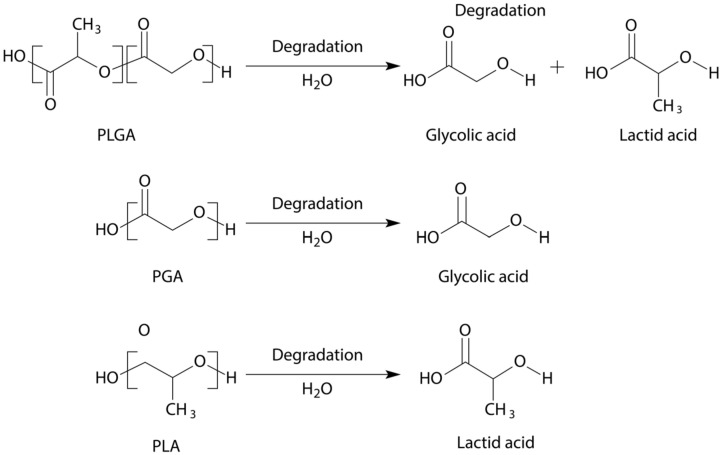
Degradation of PLGA (**top**), PGA (**middle**), PLA (**bottom**).

PLA, PGA, and PLGA are synthetic aliphatic polyesters predominantly biodegraded via non-enzymatic hydrolysis of their ester linkages under physiological conditions approved by the FDA for drug delivery (Figure 10) [10,70]. The use of these polymers has different advantages; for example, improved patient compliance, absence of foreign-body reactions, and no need of surgical procedures to remove them [86].

PLA is an eco-friendly, hydrophobic, thermoplastic, high-strength, high-modulus material approved by the FDA for direct contact with biological fluids that degrades slower than PGA and can be produced from renewable biomass resources (e.g., corn, potato, beet). PLA degradation products are not toxic in the human body; it is hydrolyzed to its constituent α-hydroxy acid, which does not interfere with tissue healing and can be eliminated in a natural way. PLA degradation depends on its molar mass, conformation, and copolymer composition; compared to its copolymer, pure PLA shows a longer half-life [70,87,88,89]. PLA implants can adopt different forms thanks to the fact that it can be processed using different techniques (i.e., injection molding, film extrusion, film forming, blow molding, thermoforming, fiber spinning). On the other hand, PLA is also a brittle material that shows low impact strength; to overcome these issues, additives such as nanoclays, isocyanates, peroxides, and synthetic rubbers have been widely used. However, to retain the biodegradable nature of PLA, only a limited quantity of non-biodegradable additives is allowed; instead, the use of other biodegradable materials to modify PLA properties should be considered [87,88].

PLA has been widely used in the treatment of ocular diseases. For example, Yasukawa et al. [90] fabricated implantable scleral plugs to treat cytomegalovirus (CMV) retinitis. Results indicate that the device does not need to be removed since PLA completely degraded in a period of months after implantation, giving way to a natural healing process where the wound was replaced with connective tissue. During the 24-week study, there was no retinal detachment, no inflammatory signs, and no abnormalities in the retinal tissue. However, little retinal toxicity and the infiltration of fibroblasts, neutrophils, and multinucleated giant cells inside the plug due to the matrix pores created in the degradation process were observed. In this work, the properties of PLA were modified, changing the molar mass of the polymer and modifying the lactide to glycolide ratio of the copolymer.

In other studies, disc-shaped PLA-intrascleral implants were evaluated. For example, a device designed for sustained intraocular delivery of betamethasone phosphate (BP) was developed by Okabe et al. [91]. In this work, after releasing BP during an eight-week period, no significant inflammation at the implantation sites or histological abnormalities were found. It also was observed that 16 weeks after implantation, no traces of PLA remained in the eye, meaning that the implant reached complete degradation. PLA disc-shaped devices have also been loaded with TA. Kim et al. [92] reported on an intrascleral implant with one side coated with a high molar mass PLA to produce a unidirectional drug release process. Toxicity and biocompatibility tests performed using rabbit eyes showed no significant inflammatory response at implantation sites, no abnormal findings in retinas during the observation time, and no histological abnormalities after 12 weeks of implantation. Regarding the device, after an eight-week inspection, it was found that the implant was well preserved. However, four weeks later, some serious cracks were found in the bottom and lateral sides of the device. TA-loaded PLA discs were also successfully used in a rabbit model of experimental uveitis. TA implant was found to be effective in suppressing inflammation for at least four weeks [93]. In both previously mentioned studies, the absence of significant abnormalities, inflammation, and/or other negative body responses to foreign objects confirmed PLA biocompatibility and low toxicity characteristics.

On the other hand, although PGA is a biodegradable semicrystalline polymer, its inherent properties limit its potential for clinical use. PGA poorly solubilizes in common polymer solvent; its synthesis involves the use of toxic solvents, which produces unsafe traces that may react with the drug or the tissue, and it is highly sensitive to hydrolysis, causing it not to be a suitable carrier in controlled drug delivery systems. Besides, it cannot be molded into films, rods, or capsules by means of any solvent or melting techniques. However, its monomer can be copolymerized with lactide, giving way to the most widely investigated biomaterial so far known for drug delivery [70,94,95].

This material is called PLGA, which is synthesized through random ring-opening copolymerization of the cyclic dimers of glycolic acid and lactic acid. Consecutive monomeric units are linked together by ester bonds. PLGA is soluble in diverse solvents including acetone, ethyl acetate, tetrahedron, and chlorinated solvents. PLGA biodegradability can be manipulated by altering its composition; the higher the glycolide content and/or the lower the molar mass, the faster the degradation rate. Therefore, materials with degradation times ranging from weeks to years can be easily obtained, consequently affecting the drug delivery system performance (e.g., rate, degree) [10,90,95,96]. FDA-approved PLGA for human applications, due to its biocompatibility and biodegradation properties, can be processed into almost into any shape and size, and can encapsulate molecules of practically any size, which turns it into the most widely used biodegradable polymer [97,98].

A wide number of laboratory investigation works using PLGA-based devices are reported in the literature, evidencing the biocompatibility and biodegradability of this polymer. For example, Souza et al. [99] performed a study on New Zealand white rabbits using Tacrolimus-loaded PLGA implants (TC-PLGA). The use of these devices showed no abnormalities during the study, and no signs of inflammation or any retinal hemorrhage or detachments. In addition, ophthalmic examination revealed no evidence of toxic effects during the six weeks of the trial, in which 99.997% of the drug was released. Peng et al. [100] evaluated the biocompatibility and biodegradability of PLGA 50/50 (i.e., 50% PLA, 50% PGA) microfilms used as subconjunctival implants in New Zealand white rabbits. An in vitro degradation test showed a directly proportional increase of water absorption and immersion time after a week, which accelerates hydrolysis and leads to remarkable drop in the molar mass, resulting in a faster degradation of the polymer. In addition, an in vivo study demonstrated the biocompatibility and non-toxicity of the implant since no evidence of inflammation, fibrosis, or scarring was evident.

PLGA implants have also been used to deliver DXM. Chennamaneni et al. [101] designed and implanted a PLGA-based device to deliver DXM to anterior and posterior chambers. Characterization tests revealed healthy tissues and no inflammation signs. Due to the inherent properties of this copolymer, even in-situ forming implants were prepared for the controlled release of DXM. PLGA molar mass and concentration ruled the polymer precipitation during solvent exchange and swelling behavior of the system, determining the inner structure of the device and, consequently, the degradation and DXM release [102]. Laboratory devices and current commercially available PLGA implants are available as described below.

**Figure 11:**
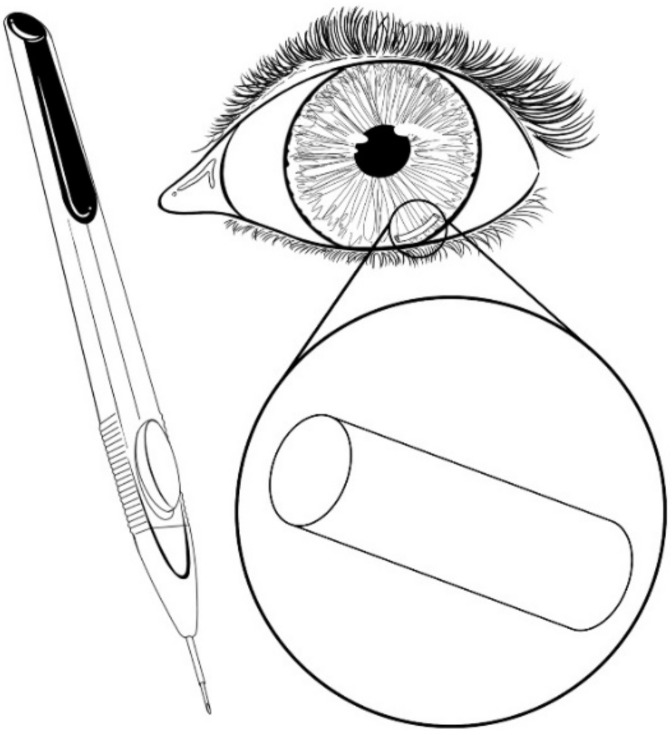
Ozurdex (PLGA-based intravitreal implant).

Ozurdex is a PLGA-based intravitreal implant (Figure 11) approved by the FDA in 2011, and was developed for the treatment of diabetic macular edema (DME), which is known to produce vision loss in patients with diabetes due to vascular leakage, fluid accumulation, and thickening of the macula. This implant is delivered intravitreally using a 22-gauge applicator by means of a shelved injection technique to produce a self-sealing wound. Ozurdex consists of a biodegradable and bioerodible matrix that releases micronized DXM to the retina and vitreous for up to six months; once inside of the eye, the copolymer degrades over time into CO_2_ and water [38,61,103,104,105,106]. According to experimental results, Ozurdex in combination with laser therapy has successfully reduced vascular leakage and retinal edema, improving visual acuity more than laser therapy alone [103]. Repetitive application of the implant also caused sustained best-corrected visual acuity and morphological central thickness improvement in eyes without the need of laser therapy [105]. Haller et al. [106] found that a single treatment produced better results than a sham procedure, reducing the risk of further vision loss and increasing the chance of improvement in visual acuity. On the other hand, Ozurdex is not recommended for eyes with post-lensectomy-vitrectomy (PLV) due to deleterious effects on the corneal endothelium. In this case, the implant migrated from the vitreous to the anterior chamber, causing eyes to develop corneal edema, requiring the implant to be removed [104]. Lowder et al. [107] evaluated the effect of this DXM intravitreal implant for the treatment of noninfectious intermediate or posterior uveitis, and found that the implant significantly improved intraocular inflammation and visual acuity, persisting for six months.

Another widely known implant is Surodex, which is a PLGA/hydroxypropyl mehtycellulose (HPMC)-based rod-shaped device loaded with DXM that is implanted into the sub-conjunctival region and the episcleral region for prolonged drug delivery to the anterior ocular tissues. This device consists of pellets that release DXM for a period of 7–10 days. Although initially it was evaluated as a treatment for postcataract surgery inflammation, it was observed that in combination with phacotrabeculectomy surgery, the use of this device offered good control of intraocular pressure with a low incidence of complications [35,108,109].

Posurdex is also a PLGA-based intravitreal implant containing DXM designed for the treatment of macular edema, uveitis, and Irvine-Gass syndrome. This device was evaluated using different drug loads (350 to 700 g), showing that higher doses of DXM are more effective at treating macular edemas. Posurdex-like devices containing 1000 g of DXM were evaluated in the treatment of inflammatory diseases in the posterior segment of the eye. Devices were implanted via pars plana into the vitreous of rabbits, showing no-n-toxic effects [110].

Finally, Allergan conducted a clinical study using an intravitreal PLGA-based implant loaded with brimonidine to prevent apoptosis of photoreceptors and/or retinal pigment epithelium with satisfactory results [111].

## 10. Materials Trends in BI

It is clear that PGA, PLA, PLGA, and PCL constitute the base of biodegradable systems. However, polysaccharides and proteins appear as novel ideal candidates for biodegradable drug delivery systems due to their renewable nature, biodegradability, biocompatibility, low immunogenicity, and thermal and pH stability under physiological conditions [112,113].

In this regard, hyaluronic acid, cellulose, gelatin, collagen, alginic acid, xantham gum and, chitosan were successfully used to improve drug delivery in the treatment of eye diseases, mainly as drug vehicles, eye drops thickening agents, surgical materials, or additives to improve contact time, permeability, and ocular absorption. However, chitosan (CS) is so far the most explored biopolymer due to its inherent characteristics, one of the most remarkable of which is its mucoadhesiveness, which enhances its ocular bioavailability [97,114,115]. For example, in 2014, Manna et al. [116] developed CS/PLA-based methotrexate (MTX) intravitreal micro-implants to treat primary intraocular lymphoma. The use of a CS as a polymer matrix instead of traditional materials such as PLA, PGA, and PLGA helped to reduce local toxicity derived from a very slow degradation process and allowed a better blending with hydrophilic drugs (e.g., MTX). Results of the in vitro study indicated that the device was able to supply MTX for a period of more than 50 days without causing any toxicity. By 2018, Manna et al. [117] improved their previous design fabricating CS-MTX micro-implants coated with different combinations of PLGA-PLA to improve the duration of MTX release. A drug release mechanism was controlled varying the PLA:PLGA ratio as well as PLA molar mass. These implants showed the potential to serve as a platform for controlled release of hydrophilic drugs for the treatment of vitreoretinal diseases. CS was also used by Badiee et al. [118] to develop ocular implants for the treatment of choroidal neovascularization, which is one of the main causes of blindness around the world. Bevacizumab-loaded chitosan nanoparticles inserted in a hyaluronic acid/zinc sulfate matrix prepared via ionic gelation released the drug over two months. Using hyaluronic acid, Van Kampen et al. [119] obtained hollow cylinder-shaped prototypes via a molding process for sustained, safe, and financially viable intravitreal protein delivery to the retina. The devices delivered therapeutic proteins for 3–6 months, which suggests a possible use as intravitreal implants.

Finally, Table 6 summarizes the role of the most commonly used materials in the design of polymeric implants as well as the geometry of the device.

## 11. Conclusions

Despite advances in ocular drug delivery methods, there is still a long way to go. Although intraocular implants were shown to be effective as drug reservoirs, the delivery system, as well as the acceptance of the implant in the body, depends largely on the material from which it is manufactured. To overcome these problems, different strategies can be followed, for example, the use of molecules that allow for the tailoring of the properties of a base material, the design of new shapes different to traditional geometries that give place to devices with higher surface areas, the use of novel technologies to conform new designs, and the use of so far non-explored materials with the potential to produce ocular implants.

## Figures and Tables

**Table 1:** Effect of VA content on the crystallinity of polyethylene-vinyl acetate [20].

Sample ^a^	VA (wt%) ^b^	DOC ^c^
PEVA11	11	45.7 ± 0.7
PEVA20	20	36.7 ± 0.8
PEVA31	31	27.6 ± 0.7
PEVA35	35	13.1 ± 0.7
PEVA44	44	8.4 ± 0.8

^a^ Sample ID, ^b^ Weight content of VA provided by the manufacturer, ^c^ Degree of crystallinity (DOC) measured using wide angle X-ray scattering (WAXS).

**Table 2:** Effect of VA content on the melting point of polyethylene-vinyl acetate [21].

Sample ^a^	VA (wt%) ^b^	T_m,p_^c^ (°C)
EVA7	7	103
EVA14	14	97
EVA20	20	70
PEVA29	29	60
EVA30	30	60
EVA40	40	25

^a^ Sample ID, ^b^ Weight content of VA provided by the manufacturer, ^c^ Melting point.

**Table 3:** Effect of different segments and chain extender on the properties of PU [56].

Segment	Effect
PEG	Increases solubility and hydrolytic degradation
PTCM	Contributes to maintain mechanical properties for long periods
PCL	Improves hydrolytic degradation
GAE	Reduces enzymatic biodegradation rate
PCN	Provides high tensile strength, yields relatively low pro-inflammatory degradation products
HEMA	Confers cross-linking functionality to the polymer
DTH	Contributes to peptide degradation by products
ISO	Enhances biological activity

PTCM: polytrimethylene carbonate, GAE: glycerol α-monoallyl ether, PCN: polyhexamethylene carbonate diol, HEMA: hydroyethyl methacrylate, DTH: desamino tyrosine tyrosyl hexyl ester, ISO: 1,4:3,6-dianhydro-D-sorbitol.

**Table 4:** PIs’ relevant properties [67].

Property	Value
Elongation at break	90%
Flexibility	2.48–4.10 GPa
Young Modulus	1.3–4.0 GPa
Density	1.31–1.43 g/cm^3^
Glass transition temperature	250–340 °C

**Table 5:** PCL relevant properties [77].

Property	Units	Value or Condition
Molar mass (of repeat unit)	g·mol^−1^	114
Weight average molar mass (Mw)	g·mol^−1^	74,000
Number average molar mass (Mn)	g·mol^−1^	25,000
Intrinsic viscosity	cm^3^·g^−1^	0.9
Physical state	-	Semicrystalline
Solvents	-	Dimethylacetamide, benzene, chloroform
Degree of crystallinity	%	69
Glass transition temperature	K	201
Melting temperature	K	331
Heat of fusion	kJ·mol^−1^	8.9

**Table 6:** Geometry and role of the materials used to build polymeric implants.

Materials	Design of the Device	Drug	Role of the Materials	References
EVA-PVA	Reservoir surrounded by a membrane	Glancicovir	EVA: membrane to limit the surface area for the permeability of the medication PVA: frame that regulates the rate of drug permeability	[8,9,14,15,16,17,18]
PVA-Silicone	Tablet geometry	FA	PVA: suture tab and coating Silicone: coating	[16,17,33,34,35,36,37,38,39]
PVA	Cylinder	FA	PVA: caps to regulate drug release rate	[40]
PVA-PMMA-Silicone	Ring	Avastin	PVA: polymeric carrier to control release and stability of the drug PMMA: reservoir material Silicone: check valves for refilling	[41]
Titanium-PVA-EVA	Helix	TA	Titanium-PVA-EVA: reservoir	[14,42]
PU-CNPs-PCL		DXM	PU: base material CNPs: as additive to modify mechanical properties PCL: as soft segment to improve polymer degradation, biocompatibility and stability	[57]
PI-PVA-Silicone	Tubular	-	PI: base material PVA: membrane Silicone: adhesive plug	[63]
PCL	-	TA, 5-FU, DXM	PCL: carrier	[80,81,82]
PLA	Disc	TA	PLA: base material	[92]
PLGA-HPMC	Rod	DXM	PLGA-HPMC: base material	[35,108,109,110]

## Data Availability

The data presented in this study are available on request from the corresponding author.

## References

[1] Nayak K., Misra M. (2018). A review on recent drug delivery systems for posterior segment of eye. Biomed. Pharmacother..

[2] Radhakrishnan K., Sonali N., Moreno M., Nirmal J., Fernandez A.A., Venkatraman S., Agrawal R. (2017). Protein delivery to the back of the eye: Barriers, carriers and stability of anti-VEGF proteins. Drug Discov. Today.

[3] Rupenthal I.D. (2017). Drug-device combination approaches for delivery to the eye. Curr. Opin. Pharmacol..

[4] Gote V., Pal D. (2019). Ocular implants in the clinic and under clinical investigation for ocular disoreders. EC Ophthalmol..

[5] Mandal A., Bisht R., Rupenthal I.D., Mitra A.K. (2017). Polymeric micelles for ocular drug delivery: From structural frameworks to recent preclinical studies. J. Control Release.

[6] Raghava S., Hammond M., Kompella U.B. (2004). Periocular routes for retinal drug delivery. Expert Opin. Drug Deliv..

[7] Urtti A., Salminen L., Miinalainen O. (1985). Systemic absorption of ocular pilocarpine is modified by polymer matrices. Int. J. Pharm..

[8] Kaji H., Nagal N., Nishizawa M., Abe T. (2018). Drug delivery devices for retinal diseases. Adv. Drug Deliv. Rev..

[9] Agrahari V., Agrahari V., Mandal A., Pal D., Mitra A.K. (2017). How are we improving the delivery to back of the eye? Advances and challenges of novel therapeutic approaches. Expert Opin. Drug Deliv..

[10] Lee S.S., Hughes P., Ross A.D., Robinson M.R. (2010). Biodegradable implants for sustained drug release in the eye. Pharm. Res..

[11] Masadeh R., Obaidat R., Alsmadi M., Altaani B., Khanfar M., Alshyab R., Qaoud M. (2018). Technical insight into biodegradable polymers used in implants. Jordan J. Pharm. Sci..

[12] Tamboli V., Mishra G.P., Mitra A.K., Mitra A.K. (2012). Biodegradable polymers for ocular drug delivery. Advances in Ocular Drug Delivery.

[13] Kleiner L.W., Wright J.C., Wang Y. (2014). Evolution of implantable and insertable drug delivery systems. J. Control Release.

[14] Wang J., Jiang A., Joshi M., Christoforidis J. (2013). Drug delivery implants in the treatment of vitreous inflammation. Mediat. Inflamm..

[15] Sanborn G.E., Anand R., Torti R.E., Nightingale S.D., Cal S.X., Yates B., Ashton P., Smith T. (1992). Sustained-release Ganciclovir therapy for treatment of Cytomegalovirus Retinitis. Arch. Ophtalmol..

[16] Yasukawa T., Ogura Y., Kimura H., Sakurai E., Tabata Y. (2006). Drug delivery from ocular implants. Expert Opin. Drug Deliv..

[17] Nicholson B.P., Singh R.P., Sears J.E., Lowder C.Y., Kaiser P.K. (2012). Evaluation of fluocinolone acetonide sustained release implant (Retisert) dissociation during implant removal and exchange surgery. Am. J. Ophthalmol..

[18] Martin D.F., Parks D.J., Mellow S.D., Ferris F.L., Walton R.C., Remaley N.A., Chew E.Y., Ashton P., Davis M.D., Nussenblatt R.B. (1994). Treament of cytomegalovirus retinitis with an intraocular sustained-release ganciclovir implant: A randomized controlled clinical trial. Arch. Ophtalmol..

[19] Ratner B.D., Badylak S.F. (2015). The biocompatibility of implant materials. Host Response to Bomaterials.

[20] Wang K., Deng Q. (2019). The thermal and mechanical properties of poly(ethylene-*co*-vinyl acetate) random copolymers (PEVA) and its covalently crosslinked analogues (cPEVA). Polymers.

[21] Chalykh A.E., Stepanneko V.Y., Scherbina A.A., Balashova E.G. (2009). Adhesive proeprties fo ehtylene and vinyl acetate copolymers. Polym. Sci. Ser. D.

[22] Schneider C., Langer R., Loveday D., Hair D. (2017). Applications of ethylene vinyl acetate copolymers (EVA) in drug delivery systems. J. Control Release.

[23] Almeida A., Possemiers S., Boone M.N., De Beer T., Quinten T., Van Hoorebeke L., Remon J.P., Vervaet C. (2011). Ethylene vinyl acetate as matrix for oral sustained release dosage forms produced via hot-melt extrusion. Eur. J. Pharm. Biopharm..

[24] Rudnik E. (2019). Properties and applications. Compostable Polymer Materials.

[25] Gohil J.M., Bhattacharya A., Ray P. (2006). Studies on the crosslinking of poly(vinyl alcohol). J. Polym. Res..

[26] Baker M.I., Walsh S.P., Schwartz Z., Boyan B.D. (2012). A review of polyvinyl alcohol and its uses in cartiage and orthopedic applications. J. Biomed. Mater. Res..

[27] Smith T.J., Pearson P.A., Blandford D.L., Brown J.D., Goins K.A., Hollins J.L., Schmeisser E.T., Glavinos P., Baldwin L.B., Ashton P. (1992). Intravitreal sustained-release ganciclovir. Arch. Ophtalmol..

[28] Musch D.C., Martin D.F., Gordon J.F., Davis M.D., Kuppermann B.D., Group G.I.S. (1997). Treatment of cytomegalovirus retinitis with a sustained-release ganciclovir implant. N. Engl. J. Med..

[29] Langer R., Brem H., Tapper D. (1981). Biocompatibility of polymeric delivery systems for macromolecules. J. Biomed. Mater. Res..

[30] Bourges J.L., Bloque C., Thomas A., Froussart F., Bochot A., Azan F., Gurny R., Ben-Ezra D., Behar-Cohen F. (2006). Intraocular implants for extended drug delivery: Therapeutic applications. Adv. Drug Deliv. Rev..

[31] Olson R.J., Kolodner H., Morgan K.S., Escapini Jr H., Sevel D., Kaufman H.E. (1980). Polyvinyl alcohol as a protective coating on intraocular lenses. Arch. Ophtalmol..

[32] Yasukawa T., Ogura Y. (2010). Medical Devices for the treatment of eye diseases. Drug Deliv..

[33] Jaffe G.J., Martin D.F., Callanan D.G., Pearson P.A., Levy B., Comstock T., Group F.A.U.S. (2006). Fluocinolone acetonide implant (Retisert) for noninfectious posterior uveitis: Thirty-four-week results of a multicenter randomized clinical study. Ophthalmology.

[34] Jaffe G.J., McCallum R.M., Brandchaud B., Skalak C., Butuner Z., Ashton P. (2005). Long term follow up results of a pilot trial of a fluocinolone acetonide implant to treat posterior uveitis. Ophthalmology.

[35] Haghjou N., Soheilian M., Abdekhodaie M.J. (2011). Sustained release intraocular drug delivery devices for treatment of uveitis. J. Ophthalmic Vis. Res..

[36] Schwartz G.S., Flynn Jr H.W. (2011). Fluocinolone acetonide implantable device for diabetic retinopathy. Curr. Pharm. Biotechnol..

[37] Brumm M.V., Nguyen Q.D. (2007). Fluocinolone acetonide intravitreal sustained release device—A new addition to the armamentarium of uveitic management. Int. J. Nanomed..

[38] Habib M.S. (2018). ILUVIEN^®^ technology in the treatment of center-involving diabetic macular edema: A review of the literature. Ther. Deliv..

[39] Brady C.J., Villanti A.C., Law H.A., Rahimy E., Reddy R., Sieving P.C., Garg S.J., Tang J. (2016). Corticosteroid implants for chronic non-infectious uveitis. Cochrane Database Syst. Rev..

[40] Mruthyunjaya P., Jaffe G.J. (2007). Medidur insert technology. Retin. Phys..

[41] Molokhia S.A., Sant H., Simonis J., Bishop C.J., Burr R.M., Gale B.K., Ambati B.K. (2010). The capsule drug device: Novel approach for drug delivery to the eye. Vision Res..

[42] Kuperman B.D., Loewenstein A. (2010). Drug delivery to the posterior segment of the eye. Dev. Ophthalmol..

[43] Kane F.E., Burdan J., Cutino A., Green K.E. (2008). Iluvien (TM): A new sustained delivery technology for posterior eye disease. Expert Opin. Drug Deliv..

[44] Campochiaro P.A., Hafiz G., Shah S.M., Bloom S., Brown D.M., Bsquets M., Ciulla T.A., Feiner L., Sabates N.B., Billman K. (2010). Sustained ocular delivery of fluocinolone acetonide by an intravitreal insert. Ophthalmology.

[45] Massa H., Nagar A.M., Vergados A., Dadoukis P., Patra S., Panos G.D. (2019). Intravitreal fluocinolone acetonide implant (ILUVIEN^®^) for diabetic macular edema: A literature review. J. Int. Med. Res..

[46] Stewart S.A., Domínguez-Robles J., Donnelly R.F., Larrañeta E. (2018). Implantable polymeric drug devices: Classification, manufacture, materials and clinical applications. Polymers.

[47] Wong I., Ho C.M. (2009). Surface molecular property modifications for poly(dimethylsiloxane) (PDMS) based microfluidic devices. Miicrofluid Nanofluid.

[48] Pinto S., Alves P., Matos C.M., Santos A.C., Rodrigues L.R., Teixiera J.A., Gil M.H. (2010). Poly (dimethyl siloxane) surface modification by low pressure plasma to imrove its characteristics towards biomedical applications. Coll. Surf. B.

[49] Chen H., Brook M.A., Shearwood H. (2004). Silicone elastomers for reduced protein adsorption. Biomaterials.

[50] Rahimi A., Mashak A. (2013). Review on rubbers in medicine: Natural, silicone and polyurethane rubbers. Plast. Rubber Compos..

[51] Lee J.H., Pidaparti R.M., Atkinson G.M., Moorthy R.S. (2012). Design of an implantable device for ocular drug delivery. J. Drug Deliv..

[52] Teoh S.H., Tang Z.G., Hastings G.W., Black J., Hastings G. (1998). Thermoplastic polymers in biomedical applications: Structures, properties and processing. Handbook of Biomaterial Properties.

[53] Subramaniam A., Sethuraman S., Kumbar S.G., Laurencin C.T., Deng M. (2014). Biomedical applications of nondegradable polymers. Natural and Synthetic Biomedical Polymers.

[54] Da Silva G.R., Da Silva Cunha A., Behar-Cohen F., Ayres E., Orefice R.L. (2010). Biodegradation of polyurethanes and nanocomposites to non-cytotoxic degradation products. Polym. Degrad. Stab..

[55] Krasowska K., Heimokswa A., Rutkowska M. (2015). Environmental degradability of polyurethanes. Thermoplastic Elastomers—Synthesis and Applications.

[56] Zhang X., Battiston K.G., McBane J.E., Matheson L.A., Labow R.S., Santerre J.P., Cooper S.L., Guan J. (2016). Design of biodegradable polyurethanes and the interactions of the polymers nad their degradation by-products within in vitro and in vivo environments. Advances in Polyurethane Biomaterials.

[57] Da Silva G.R., Da Silva Cunha A., Behar-Cohen F., Ayres E., Orefice R.L. (2011). Biodegradable polyurethane nanocomposites containing dexamethasone for ocular route. Mater. Sci. Eng. C.

[58] Pino F.C.H., Da Silva Cunha A., Orefice R.L., Ayres E., Andrade S.P., Dias C., Lima L., Moura S.A.L., da Silva G.R. (2012). Controlled release of triamcinolone actonide from polyurethane implantable devices: Application for inhibition of inflammatory-angiogenesis. J. Mater. Sci. Mater. Med..

[59] Silva Paula J., Coimbra Ribeiro V.R., Chahud F., Cannellini R., Monteiro T.C., de Lima Gomes E.C., Sol Reinach P., Veronese Rodrigues M.L., Silva-Cunha A. (2013). Bevacizumab-loaded polyurethane subconjunctival implants: Effects on experimental glaucoma filtration surgery. J. Ocul. Pharmacol. Ther..

[60] Joung Y.H. (2013). Development of implantable medical devices: From an engineering perspective. Int. Neurourol. J..

[61] Testi I., Pavesio C. (2019). Preliminary evaluation of YUTIQ^TM^ (fluocinolone acetonide intravitreal implant 0.18 mg) in posterior uveitis. Ther. Deliv..

[62] Banker A.S., Pavesio C., Merrill P. (2018). Emerging treatments ofr non-infectious uveitis. US Opthalmic Rev..

[63] Ins E.P. A Guide to Administering YUTIQ. https://yutiq.com/downloads/US-YUT-1900111%20Injection%20Brochure_single%20pages.pdf.

[64] Cai C.X., Skalak C., Keenan R.T., Grewal D.S., Jaffe G.J. (2020). Time to disease recurrence in noninfectious uveitis following long-acting injectable fluocinolone acetonide implant. Graefe’s Arch. Clin. Exp..

[65] Georgiev A., Dimov D., Spassova E., Assa J., Dineff P., Danev G., Abadie M. (2012). Chemical and physical properties of polyimides: Biomedical and engineering applications. Higher Performance Polymers—Polyimides Based—From Chemistry to Applications.

[66] Constantin C.P., Aflori M., Damian R.F., Rusu R.D. (2019). Biocompatibility of polyimides: A mini review. Materials.

[67] Comprehensive Guide on Polyimide. https://omnexus.specialchem.com/selection-guide/polyimide-pi-plastic.

[68] Lee D.J. (2015). Intraocular implants for the treatment of autoimmune uveitis. J. Funct. Biomater..

[69] Kotwal V.B., Saifee M., Inamdar N., Bishe K. (2007). Biodegradable polymers: Which, when and why. Indian J. Pharm. Sci..

[70] Christoforidis J.B., Chang S., Jiang A., Wang J., Cebulla C.M. (2012). Intravitreal devices for the treatment of vitreous inflammation. Mediat. Inflamm..

[71] Labet M., Thielemans W. (2009). Synthesis of polycaprolactone: A review. Chem. Soc. Rev..

[72] Guarino V., Gentile G., Sorrentino L., Ambrosio L. (2017). Polycaprolactone: Synthesis, properties, and applications. Encyclopedia of Polymer Science and Technology.

[73] Lance K.D., Çgood S.D., çmendes T.S., Ishikiriyama M., Chew P., Estes S., Yamada K., Mudumba S., Bhisitkul R.B., Desai T.A. (2015). In vitro and in vivo sustained zero-order delivery of rapamycin (Sirolimus) from a biodegradable intraocular device. Investig. Ophthalmol. Vis. Sci..

[74] Kim J., Kudisch K.J., Mudumba S., Asada H., Aya-Shibuya E., Bhisitkul R.B., Desai T.A. (2016). Biocompatibility and pharmacokinetic analysis of an intracameral polycaprolactone drug delivery implant for glaucoma. Investig. Ophthalmol. Vis. Sci..

[75] Bernards D.A., Bhisitkul R.B., Wynn P., Steedman M.R., Lee O.T., Wong F., Thoongsuwan S., Desai T.A. (2013). Ocular biocompatibility and structural integrity of micro-and nanostructured poly(caprolactone) films. J. Ocul. Pharmacol. Ther..

[76] Sun H., Mei L., Song C., Cui X.M., Wang P. (2006). The in vivo degradation, absorption and excretion of PCL-based implants. Biomaterials.

[77] (1999). Polymer Data Handbook.

[78] Schlesinger E.B., Bernards D.A., Chen H.H., Feindt J., Cao J., Dix D., Romano C., Bhisitkul R.B., Desai T.A. (2018). Device design methodology and formulation of a protein therapeutic for sustained release intraocular delivery. Bioeng. Transl. Med..

[79] Meng Y., Sun S., Li J., Nan K., Lan B., Jin Y., Chen H., Cheng L. (2014). Sustained release of triamcinolone actonide from an episcleral plaque of multilayered poly-e-caprolactone matrix. Acta Biomater..

[80] Borhani H., Peyman G.A., Rahimy M.H., Thompson H. (1995). Suppression of experimental proliferative vitreoretinopathy by sustained intraocular delivery of 5-FU. Int. Ophtalmol..

[81] Beeley N.R.F., Rossi J.V., Mello-Filho P.A.A., Mahmoud M.I.E., Fujii G.Y., De Juan E., Varner S.E. (2005). Fabrication, implantaion, elution, and retrieval of a steroid-loaded polycaprolactone subretinal implant. J. Biomed. Mater. Res. A.

[82] Silva-Cunha A., Ligorio Fialho S., Naud M.C., Behar-Cohen F. (2009). Poly-E-caprolactone intravitreous devices: An In vivo study. Physiol. Pharmacol..

[83] Dong X., Shi W., Yuan G., Xie L., Wang S., Lin P. (2006). Intravitreal implantation of the biodegradable cyclosporin A drug delivery system for experimental chronic uveitis. Graefe’s Arch. Clin. Exp. Ophtalmol..

[84] Shi W., Liu T., Xie L., Wang S. (2005). FK506 in a biodegradable glycolide-*co*-clatide-*co*-caprolactone polymer for prolongation of corneal allograft survival. Curr. Eye Res..

[85] Boia R., Dias P.A.N., Martins J.M., Galindo-Romero C., Aires I.D., Vidal-Sanz M., Agudo-Barriuso M., de Sousa H.C., Ambrosio A.F., Braga M.E.M. (2019). Porous poly(e-caprolactone) implants: A novel strategy for efficient intraocular drug delivery. J. Control Release.

[86] Rawas-Qalaji M., Williams C.A. (2011). Adances in ocular drug delivery. Curr. Eye Res..

[87] Farah S., Anderson D.G., Langer R. (2016). Physical and mechanical properties of PLA, and their functions in widespread applications—A comprehensive review. Adv. Drug Deliv. Rev..

[88] Semba T., Kitagawa K., Ishiaku U.S., Hamada H. (2006). The effect of crosslinking on the mechanical properties of polylactic acid/polycaprolactone blends. J. Appl. Polym. Sci..

[89] Bourges J.L., Gautier S.E., Delie F., Bejjani R.A., Jeanny J.C., Gurny R., Ezra D.B., Behar-Cohen F.F. (2003). Ocular drug delivery targeting the retina and retinal pigment epithelium using polylactide nanoparticles. Retina.

[90] Yasukawa T., Kimura H., Tabata Y., Ogura Y. (2001). Biodegradable scleral plugs for vitreoretinal drug delivery. Adv. Drug Deliv. Rev..

[91] Okabe J., Kimura H., Kunou N., Okabe K., Kato A., Ogura Y. (2003). Biodegradable intrascleral implant for ssutained introcular delivery of betamethasone phosphate. Investig. Ophthalmol. Vis. Sci..

[92] Kim Y.M., Lim J.O., Kim H.K., Kim S.Y., Shin J.P. (2008). A novel design of one-side coated biodegradable intrascleral implant for the sustained release of triamcinolone acetonide. Eur. J. Pharm. Biopharm..

[93] Shin J.P., Park Y.C., Oh J.H., Lee J.W., Kim Y.M., Lim J.O., Kim S.Y. (2009). Biodegradable intrascleral implant of triamcinolone acetonide in experimental uveitis. J. Ocul. Pharmacol. Ther..

[94] Zambon J.P., De Sá Barreto L.S., Nakamura A.N.S.E., Duailibi S., Leite K., Magalhaes R.S., Orlando G., Ross C.L., Almeida F.G. (2014). Histological changes induced by polyglycolic-acid (PGA) scaffolds seeded with autologous adipose or muscle-derived stem cells when implanted on rabbit bladder. Organogenesis.

[95] Muniswamy V.J., Raval N., Gondaliya P., Tambe V., Kalia K., Tekade R.K. (2018). Dendrimer-cationized-albumin encrusted polymeric nanoparticle improves penetration and anticancer activity of doxorubicin. Int. J. Pharm..

[96] Manickavasagam D., Oyewumi M.O. (2013). Critical assessment of implantable drug delivery devices in glaucoma management. J. Drug Deliv..

[97] Sharma A.K., Arya A., Sahoo P.K., Majumdar D.K. (2016). Overview of biopolymers as carriers of antiphlogistic agents for treatment of diverse ocular inflammations. Mater. Sci. Eng. C Mater. Biol. Appl..

[98] Makadia H.K., Siegel S.J. (2011). Poly lactic-*co*-glycolic-acid (PLGA) as biodegradable controlled drug delivery carrier. Polymers.

[99] Souza M.C.M., Fialho S.L., Souza P.A.F., Fulgencio G.O., Da Silva G.R., Silva-Cunha A. (2014). Tacrolimus-loaded PLGA implants: In vivo release and ocular toxicity. Curr. Eye Res..

[100] Peng Y., Ang M., Foo S., Lee W.S., Ma Z., Venkatraman S.S., Wong T.T. (2011). Biocompatibility and biodegradation studies of subconjunctival implants in rabbit eyes. PLoS ONE.

[101] Chennamaneni S.R., Mamalis C., Archer B., Oakey Z., Ambati B.K. (2013). Development of a novel bioerodible dexamethasone implant for uveitis and postoperative cataract inflammation. J. Control Release.

[102] Bode C., Kranz H., Siepmann F., Siepmann J. (2018). In-situ forming PLGA implants for intraocular dexamethasone delivery. Int. J. Pharm..

[103] Callanan D.G., Gupta S., Boyer D.S., Ciulla T.A., Singer M.A., Kuppermann B.D., Liu C.C., Li X.Y., Hollander D.A., Schiffman R.M. (2013). Dexamethasone intravitreal implant in combination with laser photocoaguation for the treatment of diffuse diabetic macular edema. Ophthalmology.

[104] Bansal R., Bansal P., Kulkarni P., Gupta V.K., Sharma A.K., Gupta A. (2012). Wandering Ozurdex Implant. J. Ophthalmic Inflamm. Infect..

[105] Querques L., Querques G., Lattanzio R., Gigante S.R., Del Turco C., Corradetti G., Cascavilla M.L., Bandello F. (2013). Repeated intravitreal dexamethasone implant (Ozurdex) for retinal vein occlusion. Ophthalmologica.

[106] Haller J.A., Bandello F., Belfort R., Blumenkranz M.S., Gillies M., Heier J., Loewenstein A., Yoon Y.H., Jacques M.L., Jiao J. (2010). Randomized, sham-controlled trial of dexamethasone intravitreal implant in patients with macular edema due to retinal vein occlusion. Ophthalmology.

[107] Lowder C., Belfort R., Lightman S., Foster C.S., Robinson M.R., Schiffman R.M., Li X.Y., Cui H., Whitcup S.M. (2011). Dexamethasone intravitreal implant for noninfectious intermediate or posterior uveitis. Arch. Ophtalmol..

[108] Seah S.K., Husain R., Gazzard G., Lim M.C., Hoh S.T., Oen F.T., Aung T. (2005). Use of surodex in phacotrabeculectomy surgery. Am. J. Ophthalmol..

[109] Tan D.T., Chee S.P., Lim L., Lim A.S. (1999). Randomized clinical trial of a new dexamethasone delivery system (Surodex) for treatment of post-cataract surgery inflammation. Ophthalmology.

[110] Silva G., Fialho S.L., Siqueira R.C., Jorge R., Da Silva Cunha A. (2010). Implants as drug delivery devices for the treatment of eye diseases. Braz. J. Pharm. Sci..

[111] Kuno N., Fujii S. (2012). Ocular drug delivery systems fo the posterior segment: A review. Retina Today.

[112] Jeong J., Bae S.H., Min K.S., Seo J.M., Chung H., Kim S.J. (2015). A miniaturized eye-conformable, and longterm reliable retinal prosthesis using monolithic fabrication of liquid crystal polymer (LCP). IEEE Trans. Biomed..

[113] Alvarez-Lorenzo C., Blanco-Fernández B., Puga A.M., Concheiro A. (2013). Cross linked ionic polysaccharides for stimuli-sensistive drug delivery. Adv. Drug Deliv. Rev..

[114] Dubashynskaya N., Poshina D., Raik S., Urtti A., Skorik Y.A. (2020). Polysaccharides in ocular drug delivery. Pharmaceutics.

[115] De Campos A.M., Diebold Y., Carvalho E.L., Sánchez A., Alonso M.J. (2004). Chitosan nanoparticles as new ocular drug delivery systems: In vitro stability, in vivo fate, and cellular toxicity. Pharm. Res..

[116] Manna S., Augsburger J.J., Correa Z.M., Landero J.A., Banerjee R.K. (2014). Development of chitosan and polylactic acid based methotrexate intravitreal micro-implants to treat primary intraocular lymphoma: An in vitro study. J. Biomech. Eng..

[117] Manna S., Donnell A.M., Kaval N., Al-Rjoub M.F., Augsburger J.J., Benerjee R.K. (2018). Improved design and characterization of PLGA/PLA-coated chitosan based micro-implants for controlled release of hydrophilic drugs. Int. J. Pharm..

[118] Badiee P., Varshochian R., Rafiee-Tehrani M., Dorkoosh F.A., Khoshayand M.R., Dinarvand R. (2018). Ocular implant containing bevacizumab-loaded chitosan nanoparticles intended for choroidal neovascularization treatment. J. Biomed. Mater. Res..

[119] Van Kampen E., Vandervelden C., Fakhari A., Qian J., Berkland C., Gehrke S.H. (2018). Design of hollow hyaluronic acid cylinders for sustained intravitreal protein delivery. J. Pharm. Sci..

